# Damage in a large systemic lupus erythematosus cohort from the Spanish Society of Rheumatology Lupus Registry (RELESSER) with emphasis on the cardiovascular system: a longitudinal analysis

**DOI:** 10.1136/lupus-2023-001064

**Published:** 2024-08-03

**Authors:** Irene Altabás-González, Iñigo Rua-Figueroa, Coral Mouriño, Karen Roberts, Norman Jimenez, Julia Martinez-Barrio, María Galindo, Jaime Calvo Alén, Victor del Campo Pérez, Esther Uriarte Itzazelaia, Eva Tomero, Mercedes Freire-González, Víctor Martínez Taboada, Eva Salgado, Paloma Vela, Antonio Fernandez-Nebro, Alejandro Olivé, Javier Narváez, Raúl Menor-Almagro, Gregorio Santos Soler, José Ángel Hernández-Beriain, Javier Manero, Elena Aurrecoechea, Oihane Ibarguengoitia-Barrena, Carlos Montilla, Gema Bonilla, Vicenç Torrente-Segarra, Ana Paula Cacheda, María Jesús García-Villanueva, Claudia Moriano-Morales, Concepción Fito Manteca, Nuria Lozano-Rivas, Cristina Bohórquez, José M Pego-Reigosa

**Affiliations:** 1 Rheumatology Department, Vigo University Hospital Group, Vigo, Spain; 2 IRIDIS Group (Investigation in Rheumatology and Immune-Diseases), Galicia Sur Health Research Institute, Vigo, Spain; 3 Rheumatology, Hospital Universitario de Gran Canaria Dr Negrin, Las Palmas de Gran Canaria, Spain; 4 Rheumatology, Instituto de Investigación, Galicia Sur, External Statistical Advisor, Rosario, Argentina; 5 Hospital General Universitario Gregorio Marañón, Madrid, Spain; 6 Servicio de Reumatología, Instituto de Investigación Hospital 12 de Octubre, Madrid, Spain; 7 Rheumatology, Hospital Universitario Araba, Vitoria-Gasteiz, Spain; 8 Department of Epidemiology, Vigo University Hospital Group, Vigo, Spain; 9 Department of Rheumatology, Donostia University Hospital Gipuzkoa Building, San Sebastian, Spain; 10 Rheumatology Department, Hospital Universitario de la Princesa, Madrid, Spain; 11 Rheumatology, Academic Hospital Juan Canalejo de La Coruña Cardiology Service, A Coruna, Spain; 12 Rheumatology, Hospital Universitario Marques de Valdecilla, Santander, Spain; 13 Rheumatology, Complejo Hospitalario de Orense, Ourense, Spain; 14 Department of Rheumatology, Hospital General Universitario de Alicante, Alicante, Spain; 15 Rheumatology, Hospital Regional Universitario de Málaga, Malaga, Spain; 16 Rheumatology Department, Hospital Universitario Germans Trias i Pujol, Badalona, Spain; 17 Rheumatology, Hospital Universitario de Bellvitge, Hospitalet de LLobregat, Spain; 18 Rheumatology Service, Jerez Hospital, Jerez, Spain; 19 Department of Rheumatology, Hospital Villajoyosa, Alicante, Spain; 20 Rheumatology, Hospital Insular of Gran Canaria, Gran Canaria, Spain; 21 Department of Rheumatology, Hospital Universitario Miguel Servet, Zaragoza, Spain; 22 Rheumatology, Hospital Sierrallana, Torrelavega, Spain; 23 Rheumatology Department, Hospital Universitario de Basurto, Bilbao, Spain; 24 Hospital Universitario de Salamanca, Salamanca, Spain; 25 Rheumatology, La Paz University Hospital, Madrid, Spain; 26 Rheumatology, Hospital de Sant Joan Despí Moisès Broggi, Sant Joan Despi, Spain; 27 Department of Rheumatology, Hospital Son Llatzer, Palma de Mallorca, Spain; 28 Hospital Universitario Ramon y Cajal, Madrid, Spain; 29 Department of Rheumatology, Hospital de León, León, Spain; 30 Department of Rheumatology, Universidad de Navarra-Campus Universitario, Pamplona, Spain; 31 Rheumatology, Hospital Virgen de la Arrixaca, Murcia, Spain; 32 Department of Rheumatology, Hospital Universitario Príncipe de Asturias, Alcala de Henares, Spain

**Keywords:** Lupus Erythematosus, Systemic, Cardiovascular Diseases, Autoimmune Diseases

## Abstract

**Objective:**

To assess organ damage, with emphasis on the cardiovascular system, over the different stages of the disease in a large SLE cohort.

**Methods:**

Multicentre, longitudinal study of a cohort of 4219 patients with SLE enrolled in the Spanish Society of Rheumatology Lupus Registry. Organ damage was ascertained using the Systemic Lupus International Collaborating Clinics/American College of Rheumatology Damage Index (SDI). We longitudinally analysed SDI (globally and for each domain) over time only in the 1274 patients whose dates of damage events had been recorded.

**Results:**

During the first year after diagnosis of SLE, 20% of the 1274 patients presented with new damage manifestations. At years 2 and 3, new damage was recorded in 11% and 9% of patients. The annual percentage of patients with new damage after year 5 decreased to 5%. In the first year with the disease, most damage was accumulated in the musculoskeletal, neuropsychiatric and renal systems; in later stages, most damage was in the musculoskeletal, ocular and cardiovascular systems. Considering ‘cerebrovascular accident’ and ‘claudication for 6 months’ as cardiovascular items, the cardiovascular system was the second most affected system during the early stages of SLE, with 19% of the patients who presented with damage affected at first year after diagnosis. During the late stages, 20–25% of the patients presenting with new damage did so in this modified cardiovascular domain of the SDI.

**Conclusions:**

New damage occurs mainly during the first year following diagnosis of SLE. Cardiovascular damage is relevant in both the early and the late stages of the disease. Strategies to prevent cardiovascular damage should be implemented early after diagnosis of SLE.

**WHAT IS ALREADY KNOWN ON THIS TOPIC**

- Damage is an important outcome in patients with SLE. It helps to stratify patients according to their prognosis.
- Information on how damage appears over the course of the disease is lacking.

**WHAT THIS STUDY ADDS**

- New damage in patients with SLE is more frequent at earlier stages of the disease.
- The musculoskeletal and cardiovascular systems are the main contributors to cumulative damage in early SLE.

**HOW THIS STUDY MIGHT AFFECT RESEARCH, PRACTICE OR POLICY**

- Strategies to prevent damage should be implemented early after diagnosis of SLE, with emphasis on prevention of musculoskeletal and cardiovascular damage.

## Introduction

 SLE is a multisystemic autoimmune disease characterised by a complex and diverse clinical presentation. It occurs more frequently in young women of childbearing age and can affect all ethnicities. The reported prevalence rates of SLE vary between studies, depending on such factors as study design, sex, age, geographical area and ethnicity.^1^ According to EPISER-2016, a population-based multicentre cross-sectional study by the Spanish Society of Rheumatology, the estimated prevalence of SLE in the adult population of Spain was 0.21%.^2 3^

The most recent studies on mortality in SLE show no improvement in survival in the last two decades.^4 5^ Therefore, there is an unmet need in this field, and new treatment strategies are required to improve patient outcomes. Prevention of damage accrual in SLE is a major concern for lupus specialists, since onset has been shown to occur early after diagnosis^6^ and is associated with higher mortality and poorer quality of life.^7 8^

Damage is an irreversible clinical feature that occurs after diagnosis of SLE. To be defined as such, it must have been present for at least 6 months.^9^ The manifestations of damage are not necessarily attributable only to SLE, but can also stem from comorbidities and/or drugs. Damage is evaluated using the Systemic Lupus International Collaborating Clinics (SLICC)/American College of Rheumatology (ACR) Damage Index (SDI), a validated index that measures damage accrual over time.^10 11^ An international joint initiative between SLICC, the Lupus Foundation of America (LFA) and the ACR to update the SDI is now under way.^12^

Although some reports describe damage occurring during the early stages of the disease, they involved small samples or short follow-up periods.^613^,^16^ In addition, few data are available on the timing of damage manifestations across the different SDI domains during the course of the disease. With respect to cardiovascular damage, the most widely accepted concept is that it occurs mainly during late stages of the disease.^17^

Therefore, the primary aim of our study was to assess damage in different organs/systems longitudinally over time, with emphasis on the cardiovascular system, in a large cohort from the Registry of patients with SLE of the Spanish Society of Rheumatology (RELESSER).

## Materials and patients

### Research study network

RELESSER-TRANS is a cross-sectional study in which data are collected at the time of the last medical visit (or death, when applicable). Its main objective is to describe the cumulative characteristics and comorbidities of patients diagnosed with SLE in Spain.

RELESSER was conducted by the Systemic Autoimmune Diseases Study Group of the Spanish Society of Rheumatology (EAS-SER) and involved 45 rheumatology departments. All investigators signed a written commitment before participating in the registry. RELESSER was carried out in accordance with the guidelines of the Declaration of Helsinki for research on humans^18^ and the Oviedo Convention.^19^ Data were protected in accordance with Spanish law.^20^

### Study design

We performed a national, multicentre, descriptive study of a patient cohort drawn from RELESSER-TRANS.

The current study is a longitudinal analysis of damage taking into account the date of damage events that we retrospectively collected in RELESSER-TRANS.

A detailed description of the methodology has been provided elsewhere.^21^ Briefly, a protocol was designed to gather information on approximately 400 variables per patient. The information was collected by reviewing clinical histories and inputting the data into a database. Damage manifestations were associated, when possible, with the date of their appearance. Before data collection, all investigators were encouraged to carry out a census of their patients with SLE and to complete any missing data. In order to ensure data homogeneity and quality, every item in the protocol had a highly standardised definition based on the glossaries of the most commonly used validated indices for assessment of SLE. To avoid information bias, all investigators completed a training course beforehand and had access to online guidelines on how to conduct the protocol. In order to prevent selection bias, patients were widely and homogeneously selected from across Spain. Virtually, all patients with SLE treated in Spain are referred to hospitals, thus avoiding the possibility of centre selection bias. The first patient was entered into the registry in October 2011, and electronic data collection was completed in August 2012. Then, a database review to detect any missing or inconsistent data was carried out by a professional monitor with experience in rheumatology studies. The resulting data were discussed with the principal investigators and then sent to the subinvestigators for any needed additions and corrections.

### Patients

The population of RELESSER-TRANS comprised 4219 unselected consecutive patients who fulfilled the following inclusion criteria: (a) age ≥16 years and (b) ≥4 ACR 1997 criteria for the classification of SLE^22 23^ or <4 ACR 1997 criteria, as well as a clinical diagnosis of SLE by the physician. There were no specific exclusion criteria.

On the one hand, for the description of general cohort damage, we defined the group of patients with damage as that made up of patients who presented an SDI ≥1 at some time over the course of the disease. On the other hand, as the present study is focused on the presence of damage over time, we analysed only those patients with recorded data of at least one damage event and all their dates (n=1274).

### Variables and definitions

Approximately 400 variables per patient were collected during the cross-sectional phase of RELESSER.^24^ For the current study, the information included in the analyses was as follows: demographics (age, sex and ethnicity), chronological data (data of diagnosis of SLE, disease duration, date of each damage event and date of death) and cumulative manifestations of damage (based on the SDI definitions) at the time of the physician’s last patient evaluation (or death, when applicable).

As established by the current definition of damage,^10 11^ we collected manifestations of damage observed after the diagnosis of SLE and persisting for at least 6 months.

### Statistical analysis

Our study evaluated damage manifestations in each organ/system (per SDI domain) and the temporal relationship between the time of diagnosis of SLE and the occurrence of the manifestations (longitudinal analysis).

The descriptive results were expressed as the mean (SD) for continuous variables and as the number of patients (percentages) for categorical variables.

Comparisons between the groups of patients with and without damage over the follow-up period (SDI ≥1 vs SDI=0) were carried out using the t-test for quantitative variables and the Fisher’s exact test for categorical variables.

Longitudinal analysis was performed on the 1274 patients whose dates of damage events had been recorded. It was carried out globally and per SDI domain.

We calculated the values for new damage at the different cut-off points: 1 year, 2 years, 3 years, 5 years, 10 years, 15 years and 20 years. For each cut-off point of the recently detailed follow-up and for each SDI domain, the number of new patients with new damage was calculated. Since our analysis focused on the cardiovascular manifestations of damage, we also carried out a longitudinal analysis after an SDI modification, considering ‘cerebrovascular accidents’ (neuropsychiatric domain) and ‘claudication for 6 months’ (peripheral vascular domain) as cardiovascular items. These events were included because of their cardiovascular origin, namely, thrombosis or atherosclerosis.

Statistical significance was set at p<0.05. All analyses were carried out using R Statistical Software, V.3.1.1 (R Foundation for Statistical Computing, Vienna, Austria).

## Results

The information concerning demographics, cumulative manifestations of damage (number and percentage of patients with damage, mean SDI score and mean number of SDI domains) and deaths in the cohort is shown in table 1. In order to avoid bias in their values, the numbers for these variables correspond to all of the patients (n=4219) in the RELESSER-TRANS cohort.

**Table 1:** Demographics, damage and mortality in the RELESSER-TRANS cohort*

Patient characteristics	Value
Age at diagnosis, mean (±SD), years	35.9 (15.1)
Sex, (%)	
Male	10.4
Female	89.6
Race/ethnicity (%)	
Caucasian	92
Latin American	5.3
Afro-Caribbean	0.3
Asian	0.7
Other	1.7
Disease duration, mean (±SD), months	133.2 (±105.5)
SDI score, mean (±SD)	1.1 (±1.7)
SDI score of patients with damage, mean (±SD)	2.3 (±1.8)
Patients with some damage, n/total (%)	2116/4219 (50.1)
Number of SDI domains affected, mean (±SD)†	1.8 (±1.1)
Death, n/total (%)	228/4219 (5.4)

* Data for the entire cohort (n=4219).

† Data only for those patients who presented with damage (n=2116).

SD, standard deviation; SDI, Systemic Lupus International Collaborating Clinics/American College of Rheumatology Damage Index

When we compared the general characteristics of the groups of patients with and without damage manifestation (SDI ≥1 vs SDI=0) during the course of the disease, we observed that those in the first group were more likely to be men and Caucasian. The results of this comparison are presented in table 2.

**Table 2:** Comparison of characteristics of patients with and without damage in the RELESSER-TRANS cohort

Patient characteristics	With damage (n=2116)	Without damage (n=2103)	P value
Age at diagnosis, mean (±SD), years	37.9 (16.7)	33.9 (12.9)	0.96
Sex, %			
Male	12.4	8.3	<0.001
Female	87.6	91.7	
Race/ethnicity, n (%)			
Caucasian	94.2	89.8	
Afro-Caribbean	0.2	0.4	
Latin American	4.8	5.8	<0.001
Asian	0.3	1.1	
Other	0.4	2.9	
Disease duration, mean (±SD), months	159.7 (109.9)	105.9 (93.1)	0.49
Death, n (%)	203 (9.6)	25 (1.2)	<0.001

We analysed the involvement of each SDI domain in our cohort and observed that the musculoskeletal system accumulated the most damage over the course of the disease. More detailed information about damage distribution per SDI domain at the end of follow-up is shown in table 3.

**Table 3:** Distribution of damage (per SDI domain) in the RELESSER-TRANS cohort at the end of follow-up

SDI domain	Whole cohort, n (%) (n=4219)	Patients with damage, n (%) (n=2116)
Musculoskeletal	816 (19.3)	816 (38.6)
Neuropsychiatric	539 (12.8)	539 (25.5)
Ocular	464 (11)	464 (21.9)
Cardiovascular	424 (10)	424 (20.0)
Renal	287 (6.8)	287 (13.6)
Peripheral vascular	284 (6.7)	284 (13.4)
Pulmonary	260 (6.2)	260 (12.3)
Malignancy	231 (5.5)	231 (10.9)
Mucocutaneous	231 (5.5)	231 (10.9)
Diabetes	208 (4.9)	208 (9.8)
Gastrointestinal	143 (3.4)	143 (6.7)
Premature gonadal failure	96 (2.3)	96 (4.5)

SDI, Systemic Lupus International Collaborating Clinics/American College of Rheumatology Damage Index

### Cumulative damage in the RELESSER-TRANS cohort

Mean (±SD) disease duration since diagnosis of SLE was 133.2 (±105.5) months. Damage was observed in 2116 (50.1%) patients out of the 4219 patients comprising the RELESSER-TRANS cohort. The mean (±SD) SDI score of the cohort was 1.1 (±1.7), and the mean (±SD) SDI score of the patients with damage was 2.3 (±1.8), with a mean of 1.8 SDI domains affected. More detailed information about damage in the cohort is provided in table 1.

### Description of new damage over time

For the longitudinal analysis, we analysed only those 1274 patients whose dates of damage events had been recorded. We considered early damage when it appeared during the first 5 years after diagnosis of SLE. The mean (±SD) SDI score was 0.30 (±0.62) at year 1, 0.41 (±0.73) at year 2, 0.51 (±0.86) at year 3 and 0.73 (±0.99) at year 5. The mean (±SD) number of SDI domains affected was 0.29 (±0.60), 0.40 (±0.71), 0.49 (±0.82) and 0.71 (±0.94) at the same time points, respectively.

Late damage was defined as damage that appeared 5 years after diagnosis of the disease. At year 10, the mean (±SD) SDI score was 1.22 (±1.31) and the mean (±SD) number of SDI domains affected was 1.17 (±1.25). More detailed information about new damage over time is shown in table 4.

**Table 4:** New damage over time in the RELESSER-TRANS cohort*

Year	Patients under follow-up	Patients with new damage	Annual % of patients† with new damage	SDI score, mean(SD)	Number of SDI domains affected, mean (SD)
Year 1	1251	255	20	0.30 (0.62)	0.29 (0.60)
Year 2	1221	133	11	0.41 (0.73)	0.40 (0.71)
Year 3	1199	105	9	0.51 (0.86)	0.49 (0.82)
Year 5	1178	203	9	0.73 (0.99)	0.71 (0.94)
Year 10	1113	262	5	1.22 (1.31)	1.17 (1.25)
Year 15	861	201	5	1.47 (1.40)	1.41 (1.34)
Year 20	601	144	5	1.89 (1.64)	1.81 (1.56)

* Data for those patients with all dates of damage events recorded (n=1274).

† Patients with new damage/patients under follow-up per year at risk.

SD, Standard deviationSDI, Systemic Lupus International Collaborating Clinics/American College of Rheumatology Damage Index

We observed that the percentage of patients with new damage was higher during the first year following diagnosis of SLE. A total of 225 of the 1274 patients (20%) experienced damage during the year after diagnosis of SLE. New damage was recorded in 133 patients (11%) at year 2 and in 105 patients (9%) at year 3. At year 5, new damage was recorded in 203 out of 1178 patients under follow-up, with an annual increase in damage of 9%. Five years after the diagnosis of SLE, the increase in the percentage of patients with new damage was smaller than in the previous period. During the 5–10, 10–15 and 15–20 years after diagnosis, the annual increase in new damage was 5% for each period. These findings can be seen in table 4.

We specifically analysed which domains were affected by damage during the earliest stages of the disease. The systems most affected during the first year were the musculoskeletal, neuropsychiatric, renal, cardiovascular and pulmonary systems, with 21%, 17%, 15%, 13% and 13% of patients presenting with new damage, respectively. At year 3, the musculoskeletal, ocular, renal, neuropsychiatric and pulmonary domains were the most affected, with 23%, 17%, 15%, 12% and 9% of patients presenting with new damage. At year 5, the distribution of damage among the most affected domains remained the same as in year 3.

Analysis of late damage, that is, at 10 and 15 years, showed the most frequently affected systems to be the musculoskeletal, ocular and cardiovascular systems. At the end of follow-up, we observed a higher percentage of patients with cardiovascular damage than neuropsychiatric damage. Information on new damage and the systems most affected during the different stages of the disease is presented in table 5.

**Table 5:** Distribution of new damage (per SDI domain) over time in the RELESSER-TRANS cohort*

SDI domain Patients, n (%)†	Year 1	Year 2	Year 3	Year 5	Year 10	Year 15	Year 20
Diabetes	9 (3)	4 (3)	3 (3)	10 (5)	9 (3)	11 (5)	6 (4)
Cardiovascular	33 (13)	13 (9)	7 (6)	24 (11)	41 (15)	24 (11)	23 (15)
Cancer	7 (3)	6 (4)	4 (4)	10 (5)	18 (7)	24 (11)	12 (8)
Premature gonadal failure	3 (1)	10 (7)	7 (6)	6 (3)	26 (9)	15 (7)	12 (8)
Mucocutaneous	31 (12)	10 (7)	7 (6)	13 (6)	10 (4)	9 (4)	12 (8)
Gastrointestinal	8 (3)	2 (1)	3 (3)	7 (3)	7 (3)	9 (4)	10 (7)
Neuropsychiatric	43 (16)	11 (8)	13 (12)	21 (10)	30 (11)	25 (11)	15 (10)
Ocular	20 (8)	17 (12)	18 (17)	27 (13)	56 (20)	27 (12)	27 (18)
Musculoskeletal	53 (20)	42 (30)	24 (22)	63 (30)	83 (30)	55 (25)	43 (28)
Renal	39 (15)	15 (11)	16 (15)	23 (11)	40 (15)	26 (12)	16 (11)
Pulmonary	34 (13)	11 (8)	9 (8)	18 (9)	21 (8)	21 (10)	17 (11)
Vascular peripheral	21 (8)	7 (5)	4 (4)	16 (8)	16 (6)	27 (12)	14 (9)
Patients with new damage in each period	262	139	109	211	274	219	151

* Data for those patients with all dates of damage events recorded (n=1274).

† Patients with new damage in a specific domain/patients with new damage in that period.

SDISystemic Lupus International Collaborating Clinics/American College of Rheumatology Damage Index

When we analysed ‘cerebrovascular accident’ (neuropsychiatric domain) and ‘claudication for 6 months’ (peripheral vascular domain) as cardiovascular items, we observed that this modified cardiovascular system was the second greatest contributor to damage during the early stages of the disease (years 1–5). After this modification, the musculoskeletal, modified cardiovascular and renal systems were the most affected during the first year, with 21%, 19% and 15% of the patients presenting with new damage, respectively. At year 5, the main systems contributing to damage were the musculoskeletal, modified cardiovascular, ocular and renal systems, with 31%, 18%, 13% and 11%, respectively. We also observed a change in the distribution of late damage domains. At years 10 and 15, as well as at the end of follow-up, the modified cardiovascular domain was the second most frequently affected system after the musculoskeletal domain. Information on new damage distribution (per SDI domain) over time after incorporating the modified cardiovascular domain is shown in table 6.

**Table 6:** Distribution of new damage (per SDI domain) over time in the RELESSER-TRANS cohort incorporating ‘cerebrovascular accidents’ and ‘claudication for 6 months’ in the cardiovascular domain*

SDI domain Patients, n (%) †	Year 1	Year 2	Year 3	Year 5	Year 10	Year 15	Year 20
Diabetes	9 (3)	4 (3)	3 (3)	10 (5)	9 (3)	11 (5)	6 (4)
Cardiovascular	48 (18)	20 (14)	12 (11)	37 (18)	55 (20)	45 (21)	37 (25)
Cancer	10 (3)	10 (4)	7 (4)	6 (5)	26 (7)	15 (11)	12 (8)
Premature gonadal failure	7 (1)	6 (7)	4 (6)	10 (3)	18 (9)	24 (7)	12 (8)
Mucocutaneous	3 (12)	10 (7)	7 (6)	6 (6)	26 (4)	15 (4)	12 (8)
Gastrointestinal	8 (3)	2 (1)	3 (3)	7 (3)	7 (3)	9 (4)	10 (7)
Neuropsychiatric	31 (12)	6 (4)	8 (7)	11 (5)	17 (6)	8 (4)	4 (3)
Ocular	20 (8)	17 (12)	18 (17)	27 (13)	56 (20)	27 (12)	27 (18)
Musculoskeletal	53 (20)	42 (30)	24 (22)	63 (30)	83 (30)	55 (25)	43 (28)
Renal	39 (15)	15 (11)	16 (15)	23 (11)	40 (15)	26 (12)	16 (11)
Pulmonary	34 (13)	11(8)	9 (8)	18 (9)	21 (8)	21 (10)	17 (11)
Vascular peripheral	20 (8)	7 (5)	4 (4)	15 (7)	16 (6)	25 (11)	13 (9)
Patients with new damage in each period	262	139	109	211	274	219	151

* Data for those patients with all dates of damage events recorded (n=1274).

† Patients with new damage in a specific domain/patients with new damage in that period.

SDISystemic Lupus International Collaborating Clinics/American College of Rheumatology Damage Index

## Discussion

In recent years, damage has become one of the main points of interest in the assessment of patients with lupus. The main findings of our study are the presentation of damage early after diagnosis of SLE and the importance of cardiovascular damage, at both early and late stages of the disease. It is well-known that damage leads to new damage manifestations.^25^,^32^ In addition, damage is a predictor of mortality in patients with SLE, a finding that has been observed in both the early and the late stages of the disease.^730 32^,^35^ RELESSER previously provided interesting findings about this topic, as follows: damage is more marked in complete lupus than in incomplete lupus (<4 criteria)^24^; certain domains of SDI occur more frequently in juvenile SLE^36^; there are no differences in damage between European-Caucasians and Latin-American mestizos^37^; and more damage accrual was observed in patients with late-onset SLE (≥50 years old).^38^ Although more data on damage in SLE are being reported, we continue to know little about how damage accumulates over the different stages of the disease or about which systems contribute most to such damage during the early and late stages. In our study, we tried to clarify some of these points in a large multicentre cohort of patients with SLE.

The main finding of our study is that the proportion of patients with new damage is higher during the early stages of the disease, particularly in the first year after diagnosis. While some studies assessed the incidence of damage manifestations over time, most involved smaller samples than ours and presented discordant results.^1634 35 39^,^41^ Urowitz *et al*^6^ assessed SLICC inception (<15 months after diagnosis of SLE) in a cohort of 298 patients and found that nearly half of them developed damage 5 years after diagnosis. Higher rates of damage accrual were found in two small cohorts of 202 Spanish patients and 197 Argentinian patients, with one-third in each cohort presenting with damage manifestations at year 1 and 55% and 64%, presenting with damage at year 5, respectively.^14 15^ In contrast, other authors have reported lower rates of damage accrual.^39 42^ Bandeira *et al*^42^ studied 57 patients with juvenile-onset SLE (disease onset prior to age 18 years of age) in two centres in Italy and one in Brazil and found that rates of damage accrual increased over time, with a mean modified SDI score (incorporating growth failure) ranging from 0.1 at 1 year to 0.8 at 3 years and to 1.5 at 5 years. Lower damage rates were reported in a cohort of 158 patients from Norway, with only 3% presenting with damage during the first year after diagnosis of SLE.^39^ Chambers *et al*^40^ analysed 232 patients treated by the SLE clinic at the University College London Hospital and found intermediate values of damage accrual, with 10% and 33% of the patients accruing some damage at years 1 and 5, respectively. In summary, information on damage accrual in the literature is discordant. Our finding that 20%, 9% and 9% of patients who presented with new damage did so at years 1, 3 and 5, respectively, is in line with the results from some of the aforementioned cohorts, although they clearly differ from those of the Norwegian cohort. The authors suggest that the lower rates may be due to a bias in the retrospective collection of the SDI data. On the other hand, our cohort is much larger than those of the other studies. Moreover, since the dates of damage events for all the patients included in our longitudinal analysis were recorded, such bias is unlikely. Overall, it seems that two factors associated with damage, namely, higher lupus activity^29 43^ and need for more aggressive therapy including higher glucocorticoid doses,^14 44^ play an important role in damage accrual during these early stages of the disease.

In our study, the annual percentage of patients under follow-up with new damage decreased to 5% after year 5. It is important to note that these data were calculated over the total of patients under active follow-up at each time point; therefore, bias due to loss to follow-up is overcome. Yee *et al*^31^ also reported a reduction in that rate over time in a British inception cohort of 382 patients with SLE followed up for up to 21 years. It is likely that control of SLE activity and the establishment of adequate therapeutic strategies based on the early use of glucocorticoid-sparing agents (eg, methotrexate or belimumab) contributed to lower rates of damage accrual during the later stages of the disease.

Regarding the distribution of new damage per system, we found that the musculoskeletal, neuropsychiatric, renal and cardiovascular systems were those that accumulated more damage during the initial years following the diagnosis of SLE. During later stages of the disease, new damage manifestations occurred more frequently in the musculoskeletal, ocular and cardiovascular systems. Development of organ-specific damage over time is an aspect of the disease that received little attention. Nossent *et al*^16^ analysed a multinational European inception cohort of 200 patients with SLE followed for up to 5 years and found that the main early damage was restricted to the musculoskeletal, neuropsychiatric, cardiovascular and renal domains of the SDI. These findings differ little from ours, which showed that the musculoskeletal system was involved in a high percentage of patients with SLE. Specifically, 80% of the RELESSER patients had arthritis at some point during the course of the disease.^45^ Regarding the renal and neuropsychiatric systems, these were frequently affected at diagnosis of SLE or during the early stages of the disease. However, their involvement usually required more intensive treatment, including higher doses of glucocorticoids and an immunosuppressant such as cyclophosphamide, both of which are associated with damage.^46^ Thus, it is not surprising that those are the systems that accrue more damage early after diagnosis.

The presentation of cardiovascular damage over time deserves special attention. In our longitudinal analysis, we observed the importance of early-onset cardiovascular damage. In fact, when we carried out the longitudinal analysis, modifying the SDI to include ‘cerebrovascular accidents’ and ‘claudication for 6 months’ as a modified cardiovascular system, this system gained importance, becoming the second most affected system since the first years of disease. Although this finding contrasts with the results of some seminal works, it is in line with other recent studies. In addition to reporting a 50-fold higher risk of cardiovascular disease in younger patients with SLE than in healthy controls, Manzi *et al*^47^ reported a peak in this risk around 10 years after diagnosis. Another seminal study by Urowitz *et al*^17^ found that cardiovascular disease was the leading cause of later death in patients with SLE. More recently, however, several studies from across the world have reported a higher risk of cardiovascular events around the time of diagnosis.^48^,^52^ Urowitz *et al*^49^ analysed 1848 patients from the SLICC atherosclerosis inception cohort and identified 31 patients with SLE who experienced a myocardial infarction; of these, 23 experienced it prior to diagnosis of SLE or within the first 2 years of disease. A retrospective population-based cohort study in North America^48^ obtained similar results: 70 incident SLE cases with late mean onset (52 years), in 17 of which the patients experienced 23 non-fatal cardiovascular events (myocardial infarction, stroke or congestive heart failure with hospitalisation). Interestingly, the highest rates of cardiovascular events were observed during the 2 years preceding diagnosis of SLE. In their large population-based cohort study from Canada, Aviña-Zubieta *et al*^51^ found a twofold higher risk of cardiovascular damage (myocardial infarction or stroke) in patients with SLE; this was greater during the first year after diagnosis. A study from Sweden identified 126 strokes in 3390 patients with SLE from the National Patient Register, with a relative risk of ischaemic stroke more than twice that of the general population and the highest relative risk occurring within the first year after diagnosis of SLE.^49^ Finally, Garg *et al*^52^ reported the highest frequency of incident cardiovascular events at years 2 and 11 after diagnosis in their predominantly black population-based incident SLE cohort. The results of these studies highlight the impact of cardiovascular disease preceding or occurring early after diagnosis of SLE. Although, by definition, damage in SLE should occur after diagnosis, this concept is currently under review,^12^ as the persistent inflammation that can lead to early atherosclerosis could be present before the diagnosis of SLE or even before the patient meets the classification criteria for SLE. To tackle these issues, SLICC is carrying out a revision of the SDI under the supervision of the LFA and the ACR. The results will be soon published.^11^

Delays in the diagnosis of SLE could also have played a role in the results of some of these studies.^48^ Therefore, our study highlights the importance of cardiovascular damage and the need for its prevention during the earliest stages of the disease.

One of the limitations of our study is its retrospective data collection, which can result in missing items; for example, we do not have the dates of damage events for the whole cohort. Therefore, we decided to include in our longitudinal analysis only those patients whose records had at least one dated damage event during their follow-up. As it is only a descriptive analysis, predictions or associations cannot be explained by the RELESSER-TRANS Study. However, this limitation can be overcome by conducting a further study of the prospective stage of RELESSER (RELESSER-PROS is ongoing and has thus far collected longitudinal data on about 1500 patients at annual visits over a 7-year period).

Our study also has several strengths. Nowadays, damage is an area of major interest, and research needs to focus more on long-term outcomes that are meaningful to patients. Comparisons of disease components associated with early versus late damage are both interesting and clinically pertinent, as this will enable a paradigm shift in the management of these complications early in the disease course. Finally, we analysed a large sample totalling more than 1000 patients with a long follow-up period, thus making our results consistent.

In summary, it is imperative to better understand damage in patients with SLE, since any intervention aimed at preventing onset and/or progression will likely help reduce mortality, both in the early and in the late stages of the disease. Our study of a large cohort of patients with SLE demonstrates that the first year after diagnosis is crucial, with the greatest percentage of patients with damage recorded within that period. The cardiovascular system is one of the most affected during the earliest stages of the disease. As more pronounced disease activity and higher glucocorticoid doses are common features during the early stages, minimising activity via therapeutic strategies that prioritise rapid glucocorticoid dose tapering at the start of treatment should prevent damage. These strategies, as well as interventions to reduce the cardiovascular burden, should be implemented immediately after diagnosis of SLE.

## Data Availability

All data relevant to the study are included in the article.
